# Patient-reported Outcomes as a Source of Evidence in Off-Label Prescribing: Analysis of Data From PatientsLikeMe

**DOI:** 10.2196/jmir.1643

**Published:** 2011-01-21

**Authors:** Jeana Frost, Sally Okun, Timothy Vaughan, James Heywood, Paul Wicks

**Affiliations:** ^2^PatientsLikeMe Inc.Cambridge, MAUnited States; ^1^VU AmsterdamKankerNLAmsterdamNetherlands

**Keywords:** Off-label, Internet, research, patient platform, methods, online community

## Abstract

**Background:**

Evaluating a new use for an existing drug can be expensive and time consuming. Providers and patients must all too often rely upon their own individual-level experience to inform clinical practice, which generates only anecdotal and unstructured data. While academic-led clinical trials are occasionally conducted to test off-label uses of drugs with expired patents, this is relatively rare. In this work, we explored how a patient-centered online research platform could supplement traditional trials to create a richer understanding of medical products postmarket by efficiently aggregating structured patient-reported data. PatientsLikeMe is a tool for patients, researchers, and caregivers (currently 82,000 members across 11 condition-based communities) that helps users make treatment decisions, manage symptoms, and improve outcomes. Members enter demographic information, longitudinal treatment, symptoms, outcome data, and treatment evaluations. These are reflected back as longitudinal health profiles and aggregated reports. Over the last 3 years, patients have entered treatment histories and evaluations on thousands of medical products. These data may aid in evaluating the effectiveness and safety of some treatments more efficiently and over a longer period of time course than is feasible through traditional trials.

**Objective:**

The objective of our study was to examine the illustrative cases of amitriptyline and modafinil – drugs commonly used off-label.

**Methods:**

We analyzed patient-reported treatment histories and drug evaluations for each drug, examining prevalence, treatment purpose, and evaluations of effectiveness, side effects, and burden.

**Results:**

There were 1948 treatment histories for modafinil and 1394 treatment reports for amitriptyline reported across five PatientsLikeMe communities (multiple sclerosis, Parkinson's disease, mood conditions, fibromyalgia/chronic fatigue syndrome, and amyotrophic lateral sclerosis). In these reports, the majority of members reported taking the drug for off-label uses. Only 34 of the 1755 (1%) reporting purpose used modafinil for an approved purpose (narcolepsy or sleep apnea). Only 104 out of 1197 members (9%) reported taking amitriptyline for its approved indication, depression. Members taking amitriptyline for off-label purposes rated the drug as more effective than those who were taking it for its approved indication. While dry mouth is a commonly reported side effect of amitriptyline for most patients, 88 of 220 (40%) of people with amyotrophic lateral sclerosis on the drug reported taking advantage of this side effect to treat their symptom of excess saliva.

**Conclusions:**

Patient-reported outcomes, like those entered within PatientsLikeMe, offer a unique real-time approach to understand utilization and performance of treatments across many conditions. These patient-reported data can provide a new source of evidence about secondary uses and potentially identify targets for treatments to be studied systematically in traditional efficacy trials.

## Introduction

Off-label prescribing is a legal and common practice in the United States [1]. When a manufacturer develops a new medication for a particular purpose, the US Food and Drug Administration’s (FDA’s) [2] Center for Drug Evaluation and Research evaluates the drug’s efficacy and utility for that purpose before it is brought to market. However, once the drug is on the market, health care providers are free to prescribe the drug for either the FDA-approved purpose (“indication”) or any other purpose – a practice referred to as “off-label prescribing.” Across all major drug categories, it is estimated that 21% of all prescriptions are for off-label purposes [3].

Off-label prescribing has the potential to be a source of innovation in medicine. Prescribers can discover novel uses for existing medications while monitoring tolerability, safety, and effectiveness. Within their practice they can apply the insight acquired from treating one person to the next case [4]. However, prescribers may not have an adequate number of cases to distinguish between a truly meaningful effect of the drug, and either a placebo effect or the tendency for patients to improve on their own.

Off-label prescribing is often done without supporting medical evidence [1]. For the estimated 21% of prescriptions given off-label, 73% lacked scientific evidence underlying their use [3]. In many cases, patients may be subject to unnecessary, ineffective, and even harmful treatments. In the past, millions of women received prophylactic hormone treatment for the prevention of osteoporosis, for instance. However, systematic evaluation revealed no therapeutic benefit and elevated risks of cardiac damage [5]. Patients are extremely unlikely to be aware that the FDA has not evaluated the safety and efficacy of their treatment for the purpose for which they are using it.

In 2008 the FDA released a guidance document entitled “Good Reprint Practices for the Distribution of Medical Journal Articles and Medical or Scientific Reference Publications on Unapproved New Uses of Approved Drugs and Approved or Cleared Medical Devices” [2]. This guidance provides advice for industry on circulating journal article reprints that contain information on off-label use, such as for the use of modafinil in treating fatigue in multiple sclerosis (MS) or amitriptyline in treating excessive saliva in amyotrophic lateral sclerosis (ALS). Unfortunately there are a number of limitations to the application of this guidance. First, the quality of the scientific literature and the informal benchmark of acceptability vary dramatically between diseases. The most widely cited paper on the use of modafinil for the treatment of fatigue in MS, for instance, has been cited nearly 250 times but contained only 65 patients at its 9-week end point and failed to find a dose-response effect [6]. Second, the guidance requires industry to provide counterbalancing evidence. Perhaps unsurprisingly, though, there is evidence of selective reporting: many off-label trials are not published, particularly when their finding are not significant [7]. This effect is surely compounded by publication bias; that is, it is easier to publish significant findings than nonsignificant findings. Third, there are inconsistencies among medical conditions in the number of options available; off-label medication use is frequently the only option for “orphan conditions”[1]; and for many medical conditions there is no “approved” treatment. For instance, a study comparing approved drug indications against the *Diagnostic and Statistical Manual of Mental Disorders*, 4th edition, text revision (DSM-IV-TR) found that 89% of all psychiatric disorders lack approved medications for their treatment [8]. Fourth, the regulations apply only to the most visible means of promotion, and fail to address continuing medical education presentations and events, or oral responses to physicians’ questions at company-sponsored events, exhibit booths, or in “detailing” visits [9]. Finally, it has been noted that, as the guidance is not legally binding, enforcement is unlikely.

Consequently, there is a need to gather evidence to inform off-label prescribing. Unfortunately, gathering this evidence can be prohibitively expensive. Running a clinical trial, of the type needed to establish the efficacy of a drug for a new purpose, costs US $15,700 for a phase 1 trial and over US $26,000 for a phase 3 trial per patient [10]. If a drug is already being used widely off-label there may be no incentive for manufacturers to evaluate it systematically.

In this paper, we propose a new source of evidence to evaluate off-label use: patient-reported outcomes entered via an online community. An increasing number of patients are going online to access information about their health and talk to other patients about a shared condition [11]. Many patients share advice and details about their treatments and symptoms with one another and with researchers. Clinical trial researchers increasingly use the Internet for recruiting subjects, communicating with participants, and even collecting data [12]. Patient groups like the Life Raft Group for patients with gastrointestinal stromal tumor have successfully mobilized their members to study the effectiveness of investigational treatments [13]. In this work, we suggest how patients, entering outcomes within an online community, could inform how drugs are working for off-label uses by expanding the available evidence base.

To conduct this analysis we examined patient reported outcomes reported on PatientsLikeMe. PatientsLikeMe is a web-based community and research platform where patient members share details about their treatments, symptoms, and conditions, with the intention of improving their outcomes[14,15]. Patients join communities designed specifically for their condition. At the time of writing, there were 11 distinct patient communities and over 70,000 patient members. The site synthesizes members’ data into interactive reports for review. Each member sees a graphical representation of their own and others’ function, treatments, and symptoms over time and can view reports of aggregated data. The site includes an interactive treatment report for each medication and intervention that patients add to the system. The report includes dosages taken, time on treatment, and evaluations of the treatment, including perceived efficacy, side effects, and burden. These treatment reports can suggest usage patterns and effectiveness rates for drugs across different purposes.

We examined patient data for two medications that are widely prescribed off-label and currently being evaluated for new applications: amitriptyline and modafinil. We documented how patients reported using these drugs and how patient reports could inform broader understanding of these relatively well-understood medications. PatientsLikeMe hosts communities for patients with ALS, MS, depression, Parkinson's disease, fibromyalgia, and chronic fatigue syndrome. Given the high levels of fatigue, pain, excess saliva, and depression presented across these communities, many members of the site could be candidates for treatment of these symptoms.

Amitriptyline (Elavil; Merck & Company Inc, Whitehouse Station, NJ, USA) is a tricyclic antidepressant that was developed by Merck and approved in the United States in 1961. It has FDA approval for the treatment of major depression, clinical/endogenous depression, and involutional melancholia, but it is commonly used off-label for other symptoms ranging from chronic pain to bed wetting. Due to the anticholinergic effects of amitriptyline a primary side effect of the drug is dry mouth. There are 14 clinical trials involving amitriptyline that are recruiting subjects (on clinicaltrials.gov), reflecting an ongoing interest in its use. In neurological conditions such as ALS, amitriptyline has been reported informally as being used by neurologists for the treatment of depression, as well as off-label for excessive saliva, emotional lability, urinary urgency, and insomnia [16], despite an absence of trials supporting its use. Even in its indicated use, for depression, ALS guidelines state “Concerning pharmacological treatment of depression in patients with ALS, there is broad consensus among clinical experts that [selective serotonin-reuptake inhibitors] and [tricyclic antidepressants] are helpful, but there have been no controlled clinical studies of these medications in ALS patients” [17]. Antidepressants like amitriptyline have been highlighted as an important target for future research into off-label drug use [18].

Modafinil (Provigil; Cephalon, Inc, Frazer, PA, USA) is a wakefulness-promoting agent first available in the United States since 1998 for approved purposes related to sleep disorders, including narcolepsy, shift-work disorder, and obstructive sleep apnea. As a wakefulness-promoting agent, it has also been investigated off-label for the treatment of fatigue in conditions including MS [19], fibromyalgia [20], chronic fatigue syndrome [21,22], and Parkinson's disease [23-25]. In the past, promotion of the drug for these off-label purposes by the manufacturer has resulted in warnings and fines from the FDA [2]. A recent review [19] of the MS literature assessing the use of modafinil for the treatment of fatigue in MS considered it a “reasonable therapeutic option” but cautioned that trials to date have been small (total N of the literature = 308 patients), unblinded, and with only short-term follow-up (median follow-up 12 weeks). There were some adverse events, mostly gastrointestinal, but one-third of studies failed to report adverse events at all. Similar methodological problems likewise seriously undermine existing off-label studies in other diseases.

In this study, we conducted a post hoc analysis of the prevalence of on-label versus off-label use, dosing, and perceived effectiveness and side effects for these medications. We looked at prevalence of use across the site and in specific communities. We documented purposes of use by community and the side effects they reported. Lastly, we began to look at how effectiveness varied by purpose to see whether these agents function similarly for on- and off-label indications.

## Methods

We analyzed the treatment information entered by patient members about the two drugs of interest, amitriptyline and modafinil, across five condition-based communities: MS, fibromyalgia/chronic fatigue syndrome, ALS, mood disorders (depression, bipolar disorder, and anxiety disorders), and Parkinson's disease. At the time of analysis (May 24, 2010), these communities contained 53,928 members.

**Figure 1:**
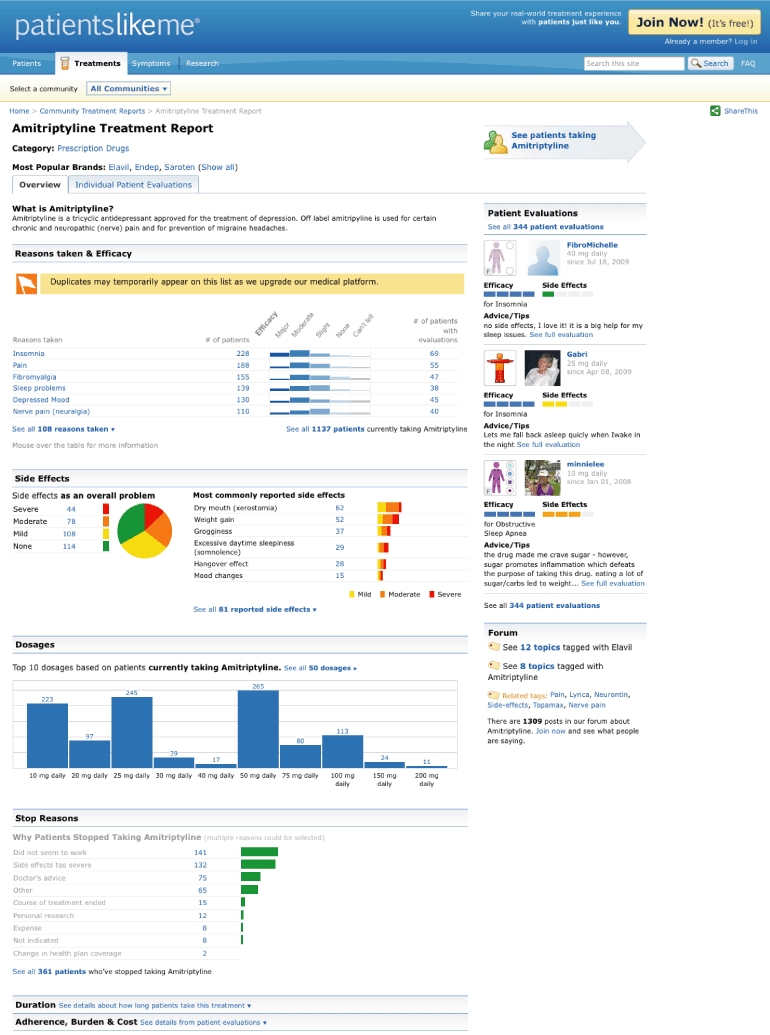
The treatment report for amitriptyline available on PatientsLikeMe. This treatment report was captured after the date of analysis; therefore, the data featured do not match the data reported.

Patients complete treatment histories, including start date, the purpose for taking the treatment, dosage (with available dosages according to the Multum database [Cerner Multum, Denver, CO, USA] prompted as the most likely response options), dates of dosage change, and stop date. Members can add more than one treatment history to indicate repeated trials of a treatment. In addition to their treatment history, members may complete evaluations for each treatment, entering side effects, severity of side effects (none, mild, moderate, or severe), burden (difficulty of being on treatment: not at all, a little, somewhat, or very), and perceived effectiveness (can’t tell, none, slight, moderate, or major). In both the treatment reports and the evaluations (See Figure 1), users are prompted to use a curated vocabulary of side-effect and purpose terms, but may enter their own natural language if they wish. In order to aggregate data across the patient-entered vocabulary, patient-generated symptom and side-effect symptom terms were coded using the Medical Dictionary for Regulatory Activities (MedDRA MSSO, Chantilly, VA, USA).

## Results

### Modafinil

Across the five communities, there were 1948 treatment histories for modafinil: 1316 described current treatment at the time of analysis; therefore, 1316 of 53,928 (2%) of all members reported currently taking modafinil.

Modafinil use was most prevalent in the MS community, where there were 1565 reports for 17,820 members (6%), followed by Parkinson's disease (75/4789, 1%) and mood communities (136/14,483, 1%). Purposes were reported in 1755 of 1948 (90%) modafinil treatment histories (see Table 1, n = 1755). Overall, only 34 of 1755 (less than 1%) of members reported taking modafinil for an approved purpose (narcolepsy and excessive daytime sleepiness resulting from sleep apnea; see Figure 1). Rather, the majority of users reported taking modafinil to treat other issues, including general fatigue (1201/1755, 68%) and excessive daytime sleepiness or tiredness arising from their condition (288/1755, 16%); less common purposes included “brain fog” (61/1755, 3%) a patient vocabulary term for having difficulty concentrating, and cognitive impairment (29/1755, 2%).

When purposes were viewed by MedDRA system organ class (SOC) terminology, modafinil was most commonly used to treat purposes that fall within “general disorders and administration site conditions” (1277/1755, 73%) followed by “nervous system disorders” (415/1755, 24%). No other category accounts for more than 1% of responses.

There were 726 treatment evaluations written about modafinil at time of analysis and 383 side-effect reports. The most common side effects reported fell into the MedDRA SOC “nervous system disorders” (134/383, 35%) and “general disorders and administration site conditions” (100/383, 26%). Looking at individual side effects, jittery feeling (68/383, 18%), dry mouth (60/383, 16%), and anxiety (46/383, 12%) were the most commonly reported.

In these evaluations, most users (532/726, 72%) rated the effectiveness of modafinil in the highest response categories: either “major” or “moderate” (see Table 2). These effectiveness ratings did not vary by purpose. There was a slight tendency to rate the drug as more effective for some off-label purposes, such as a treatment of MS, than for sleepiness. There was only one evaluation in the system for an approved purpose, specifically narcolepsy.

**Table 1:** Purposes reported by 10 or more users for modafinil

Purpose reported	MedDRA LLT code^a^	Number reporting (n = 1755)	%
Fatigue^b^	10016256	1201	68.43%
Excessive daytime sleepiness^b^	10015595	262	14.9%
Brain fog	10016876	61	4%
Mood	10027945	26	2%
Sleepiness	10041014	26	2%
Cognitive impairment	10009846	29	2%
Narcolepsy and sleep apnea	10028713; 10040975	24	1%
Problems concentrating	10003729	20	1%
Multiple sclerosis	10028245	23	1%

^a^ Medical Dictionary for Regulatory Activities lower-level term.

^b^ 68% of users reported taking the drug to treat fatigue and another 14% excessive daytime sleepiness, such that most users appear to have taken the drug for related purposes.

**Table 2:** Effectiveness ratings for modafinil and amitriptyline

Effectiveness rating	Modafinil (n = 726)		Amitriptyline (n = 590)	
	# Reporting	%	# Reporting	%
Can’t tell	30	4%	36	6%
No effect	23	3%	86	15%
Slight	141	19%	167	28%
Moderate	268	37%	201	34%
Major	264	36%	100	17%

### Amitriptyline

There were 1,394 treatment reports for amitriptyline: 865 of the total 53,928 patient members reported currently taking the drug (2%).

ALS, although a small community, had the highest rate of use. At the time of analysis, 228 of 4060 (6%) ALS patients in the community reported having taken the drug and 178 of the 4060 (4%) ALS patients stated they were currently taking amitriptyline. In 1197 of the 1394 (86%) treatment reports, patients reported a purpose (see Table 3). Off-label uses were much more commonly reported than the on-label purpose. In 104 of 1197 reports (9%), patients reported taking amitriptyline for the approved use of depression; most commonly, patients reported taking it for insomnia and other sleep problems (321/1197, 27%) or pain (197/1197, 17%). Examining purposes at the SOC level found that members reported using amitriptyline to control complaints in a variety of systems, including nervous system disorders (544/1197, 45%), musculoskeletal and connective tissue disorders (115/1197, 10%), and gastrointestinal disorders (103/1197, 9%). Psychiatric disorders, more broadly, accounted for only 208 (17%) of the 1197 reported purposes. One purpose of note was in ALS, where 88 of 220 (40%) patients took the drug for the purpose of treating a symptom of their condition, excess saliva.

Overall, there were 270 side-effect reports of amitriptyline in the system. The most commonly reported side effects were related to feeling sleepy (reported 126 times in 270 reports, 46%), including “grogginess/sleepiness/drowsiness” (reported 56 times in 270 reports), “daytime sleepiness” (reported 34 times), and “feeling groggy” (reported 36 times in 270 reports). The second most common side effect was dry mouth (reported 78 times in 270 reports, 29%) and third was weight gain (60/270, 22%).

In this example, there were 70 effectiveness ratings for the approved purpose of depression and 520 effectiveness ratings for off-label purposes (see Table 2). The ratings for off-label purposes were higher than for depression: 28 of the 70 (40%) respondents taking it for the prescribed purpose of depression rated it as having either a major or moderate efficacy in comparison to 273 of 520 (52%) taking it for off-label uses.

**Table 3:** Most common purposes reported for taking amitriptyline: purposes reported by 10 or more users are listed (n = 1197 purpose reports by 1394 users). The reasons people reported taking the drug vary widely.

Purpose reported	MedDRA LLT code^a^	Number reporting (n = 1197)	%
Insomnia/sleep problems	10022437	321	26.8%
Pain	10033371	197	16.5%
Depression	10012378	104	8.7%
Fibromyalgia	10048439	90	8%
Excess saliva	10021677	88	7%
Nerve pain	10029181	83	7%
Emotional lability	10014555	37	3%
Migraine headaches	10027602	37	3%
Anxiety	10002855	36	3%
Headaches	10019211	24	2%
Mood disorder	10027945	21	2%
Muscle pain	10028322	17	1%
Restless legs syndrome	10038741	14	1%
Migraine	10027599	13	1%
Fatigue	10016256	11	1%
Amyotrophic lateral sclerosis	10052889	10	1%
Stiffness/spasticity	10041416	10	1%

^a^ Medical Dictionary for Regulatory Activities lower-level term.

## Discussion

Using an online patient community, PatientsLikeMe, we identified that only less than 1% of nearly 2000 patients taking modafinil and 9% of nearly 1400 patients taking amitriptyline reported taking each drug for purposes approved by the FDA. In both cases, patients subjectively reported the effectiveness for off-label uses as either higher than or comparable to approved indications. Many patients used some of the most common side effects reported for amitriptyline, including sleepiness, as their purpose for taking the drug, such as the treatment of insomnia.

We were surprised to find that in two relatively well-understood drugs, the vast majority of uses were off-label. Our analysis may indicate that off-label prescribing is even more common in certain patient populations. In terms of patient-reported effectiveness, the data suggest that amitriptyline could be more efficacious for off-label uses than for FDA-approved uses. Further study of newer, less commonly used for off-label purposes, would provide a more complete understanding of the value of patient-reported outcomes in this area.

One advantage of collecting treatment information through an online community is the ability to reach a large population of users at relatively little marginal cost. As the Internet becomes more accessible, an increasingly diverse population is online and joining online communities for support with health problems [11]. By gathering experiences directly from patients, researchers can elicit new types of data not recorded systematically through routine clinical practice, and which would be unlikely to attract funding for traditional clinical trials. In fields of study where self-reported data are acceptable, the Internet offers a unique vehicle to collect vast quantities of data far more effectively than traditional studies permit. This is particularly true for ongoing monitoring of patient safety and serious adverse events. Toward this end, PatientsLikeMe is developing its pharmacovigilance platform to provide a constant stream of safety data to manufacturers and the FDA, which can serve as an ongoing phase 4 study of pharmaceutical products.

However, there are significant challenges associated with collecting patients’ outcome data for post hoc analysis. Members of an online community visit the site on their own schedule and contingent upon their own needs. While a website may prompt users for specific information at timed intervals, members ultimately have the choice of when and whether they will add data. Members may add data only when they feel strongly about a treatment, leading to a substantial reporting bias. In this light, unblinded studies like ours might consider different outcome metrics of primary importance and rely on markers of perceived treatment effectiveness such as discontinuation rates, adherence and side effects, rather than self-reported measures of effectiveness, which can be highly susceptible to placebo effects.

Yet there are other limitations. Within the group of registered patients in a community, patients may not report information completely. An unknown proportion may be taking the drug but fail to report it or its effects. Among those who have taken the treatment, only a certain subset completed an evaluation of the drug, and for the most part they evaluated it at only one timepoint. It is hoped that prompts and improved user interface designs, along with more contextual reports and research studies (such as this one), will increase the value to patients and in turn motivate users to enter more information.

In addition, we have a lower level of confidence than in clinical trials that a registered “patient” in our system has had a specific condition diagnosed, that the user is taking the medication as prescribed, or that the patient’s experience is tempered by an unreported comorbidity. There is the potential in the future to ask clinicians to verify diagnoses and to use records from the pharmacies or eHealth technology to validate patient-reported behavior, but this will require significant research to address issues of consent and coding requirements to ensure privacy. In the meantime we believe that the scale, scope, and cost to execute such studies outweigh, or at the very least, mitigate, these limitations.

Due to the architecture of the PatientsLikeMe system, we included only a handful of medical communities and possibly incomplete patient experiences. However, plans are underway to significantly expand the number of communities and allow for multiple comorbidities to be collected, thereby increasing the scope, quality, and representativeness of future studies.

Finally, when collecting data from patients online, there is the distinct possibility of more egregious misrepresentation – namely, that users are not who they appear to be. Patients on the site could be falsifying their identities entirely. While this is always possible, certain Internet platforms may be at higher risk for these gross inaccuracies than others. In many websites built specifically to collect medication ratings from patients, users enter minimal information about themselves before entering treatment evaluations, thus lowering the barrier for misrepresentation. PatientsLikeMe, as a community based on ongoing interaction and a reputation built upon a time-based health profile, may be less susceptible to flagrant misrepresentation.

### Conclusion

There are stated methodologies to evaluate the safety and efficacy of drugs for a specific purpose before they are brought to market. Once approved, these drugs are being used to treat a wide variety of symptoms and conditions. In many cases, this is a legitimate and ultimately positive use for an existing agent, yet all too often there is no way to establish evidence or monitor patient safety.

At the moment, providers often rely on peer-reviewed literature to inform treatment choice. But critics note that “attempting to use peer-reviewed literature for a purpose for which it is so ill suited is likely not only to fail to adequately regulate off-label use but also to degrade the quality of peer-reviewed literature” [26], suggesting there is a need for other ways to evaluate off-label prescribing. Online patient platforms, as a repository for patient-reported outcomes, provide an opportunity to create new methods to study the effect of these drugs after they have reached the market. Evaluating evidence from multiple sources, including peer-reviewed literature and online communities, could provide converging evidence about effectiveness. Online communities are in the unique position to capture and present information of particular relevance to other patients who are considering taking a drug.

Off-label prescribing is a common practice, but outcomes associated with it are routinely understudied, which sometimes leads to wasteful treatments and even harmful effects. We propose that patients, sharing their data online, can provide relevant, timely information to fill these gaps in knowledge.

## Abbreviations

ALS: amyotrophic lateral sclerosis
DSM-IV-TR: Diagnostic and Statistical Manual of Mental Disorders, 4th edition, text revision
FDA: Food and Drug Administration
MedDRA: Medical Dictionary for Regulatory Activities
MS: multiple sclerosis
SOC: system organ class
